# Research Progress on the Mechanism of Persistent Low-Level HBsAg Expression in the Serum of Patients with Chronic HBV Infection

**DOI:** 10.1155/2022/1372705

**Published:** 2022-04-13

**Authors:** Jie Wu, Yu Yu, Yuzhu Dai, Yingjie Zhang, Jun Cheng

**Affiliations:** ^1^School of Laboratory Medicine, Bengbu Medical College, Bengbu 233000, China; ^2^Department of Clinical Research, The 903rd Hospital of PLA, Hangzhou 310013, China

## Abstract

Among HBV-infected persons, there is a group of people with hepatitis B surface antigen (HBsAg) showing persistently low levels of expression. The production of low-level HBsAg does not mean a good outcome of chronic HBV infection. Patients still have virus replication and sustained liver damage, and they have the potential to transmit the infection. This risk poses a challenge to clinical diagnosis and blood transfusion safety and is a major concern of experts. However, the mechanism behind persistent low-level HBsAg expression in serum is not completely clear, and complete virus clearance by the host is vital. In this review, we summarize the research progress on the mechanism behind low-level expression of HBsAg in patients with chronic HBV infection in recent years.

## 1. Introduction

HBV infection poses a serious threat to human health and social public health security. There are approximately 257 million people with chronic HBV infection around the world, but the prevalence of HBV varies greatly in different regions [1]. HBV belongs to the hepatophilic DNA virus family, with a total length of approximately 3200 bp, and it is comprised of the envelope, core, DNA genome, and virus polymerase [2]. The complete infectious virus particle can be visualized under an electron microscope, that is, HBV spherical (Danish particle). It has a host-derived outer surface lipid shell, which contains a surface antigen comprised of large (L-), middle (M-), and small (S-HBsAg) and an inner core protein called hepatitis B core antigen (HBcAg). In addition to intact virus particles, there are also smaller noninfectious subviral particles in the serum, including 17-25 nm spherical particles comprised of SHB proteins, which constitute the most abundant form, more than 10 000-fold abundant than intact infectious virus particles; another form is filamentous (or tubular) particles of variable length, comprised of SHB, MHB, and LHB proteins [3]. The pre-S1 domain of L-HBs plays a key role in the virus envelope and drives infectivity [4], and the form of the HBV particles appears to be determined by the ratio of the various HB proteins. All three viral forms can be detected in serum and are collectively referred to as HBsAg [5].

HBV serological examination, serum biochemical examination, HBV DNA (hepatitis B virus load) quantification, and other auxiliary clinical diagnosis methods are important indicators for the selection of treatment and the judgment of the curative effect of antiviral therapy [6]. HBsAg is one of the key serological markers for the diagnosis of HBV infection, and it is also an important screening index to judge the effect of HBV treatment and evaluate the safety of blood transfusion [7]. Experts agree that when the patient's serum HBsAg is completely cleared or negative seroconversion occurs, this is an ideal endpoint for the treatment of chronic HBV infection [8]. Quantification of HBsAg (qHBsAg) may be helpful for staging HBV infection and predicting its response to antiviral therapy [9]. According to the results of serology, biochemistry, virology, and other assistant examinations of chronic hepatitis B (CHB) infection, it can be divided into a CHB-carrying state (immune-tolerant phase, IT), inactive HBsAg-carrying state (low-replicative phase, LR), HBeAg- (hepatitis B e antigen-) positive CHB (immune clearance phase, IC), and HBeAg-negative CHB (reactivity period, HBeAg-negative hepatitis, ENH) [10].

At present, more and more studies have found that some patients with chronic HBV infection have sustained low-level expression of HBsAg, which shows an inactive HBsAg-carrying state, that is, most of them are chronic asymptomatic HBV carriers (ASCs) [11], which is different from HBeAg-positive immune-tolerant phase. The clinical manifestations of HBeAg-positive immune-tolerant phase are generally strong HBsAg, HBeAg, and HBcAb positive, and the level of HBV-DNA is also high. In addition, the sustained low-level expression of HBsAg is also easily confused with occult hepatitis. Under the selection pressure of endogenous (host immunity) or exogenous (vaccination and antiviral treatment) immune suppression, the number of chronic HBV-infected people with low-level HBsAg expression has shown an upward trend. There is low replication of HBV DNA in people with low-level HBsAg expression [12], which has a potential risk of transmission; therefore, studying the expression and formation mechanism of low-level HBsAg in patients with chronic HBV infection may provide new ideas for further preventing HBV transmission and effectively clearing low-level HBsAg.

For low-level HBsAg expression, many people think it is a kind of occult hepatitis, so what is the difference between it and occult hepatitis? Is the low level of HBsAg expression transient or persistent? What is the relationship between the formation mechanism of low-level HBsAg expression, gene mutations, and host immune clearance? Focusing on these questions, this paper summarizes the concept and the mechanisms behind low-level HBsAg expression.

## 2. Persistent Low-Level HBsAg Expression and Occult Hepatitis B Virus Infection (OBI)

According to the differences in serum contents, there are different grouping criteria for high-level HBsAg and low-level HBsAg. In a study of liver histopathological changes and related factors in patients with low-level HBsAg, Liu et al. [13] selected HBsAg at 0.05~1400 IU/ml as the low-level group. In evaluating the relationship between the serological pattern of HBsAg/anti-HB coexistence and the risk of hepatocellular carcinoma in patients with chronic HBV infection, Jin et al. [14] regarded the samples with serum HBsAg levels <1000 IU/ml as the low-level group. Lai et al. [15] also selected samples with serum HBsAg levels < 1000 IU/ml as the low-level group in a study of a multidose hepatitis B recombinant vaccine for chronic hepatitis B patients with low surface antigen levels. Yang et al. [16] defined a content of low-level HBsAg in serum as 0.05-99 IU/ml in a study of the individual and comprehensive effects of the HBsAg level and viral load on the risk of liver cancer. In the past, the content of serum HBsAg was mostly detected by enzyme-linked immunosorbent assay (ELISA), but some studies showed that there were false-positives and false-negatives in the detection of low-level HBsAg by ELISA [17]. With the continuous development of detection methods and the introduction of high-sensitivity kits, the definition of low-level HBsAg is constantly being updated. The clinical testing center of the National Health Commission of China has provided a serum standard with a low-level fixed value, the chemiluminescence microparticle immunoassay (CMIA) method replaced the traditional ELISA method for quantitative detection of serum HBsAg, low-level HBsAg was defined as serum HBsAg < 5.0 ng/ml or <10.0 IU/ml [18], which improved the detection rate of low-level HBsAg.

Occult hepatitis B infection (OBI) is a condition in which serum HBsAg is undetectable but serum and/or intrahepatic HBV DNA is detectable. OBI can be caused by self-limited acute hepatitis or in patients with CHB who reached HBsAg seroclearance, which refers to the loss of detectability of serum HBsAg with or without antibodies against HBsAg (anti-HBs) in CHB patients [19]. Mutations in the HBsAg “a” determinant lead to structural alignment of the protein, which may render HBsAg undetectable by commercial HBsAg detection kit. Mutations in the S region are associated with decreased expression of HBV surface proteins, and mutations in the preS1/preS2 promoter are frequently observed in OBI patients, which renders HBsAg undetectable [20]. The mechanism of chronic HBV infection with sustained low-level HBsAg expression is also related to mutations in the pre-S1, pre-S2, and S regions of the HBsAg gene [21, 22], indicating that there is partial crossover between low-level HBsAg population and occult hepatitis infection, but low-level HBsAg population can detect low concentrations of HBsAg in the host serum and accompanied by low or no replication of HBV DNA. At the same time, if there is a false-positive in the detection of low-level HBsAg, a confirmation test can be carried out if necessary to ensure the accuracy of the experimental results [23]. In addition, due to mutations in the HBsAg “a” determinant, S region, etc., HBsAg cannot be detected in OBI patients. When the immune function declines or receives immunosuppressive therapy, the host will not develop immune tolerance, but may lead to HBV reactivation [24]. However, when the host's immune function is compromised, the low-level HBsAg population will develop immune tolerance under the combined effect of the virus's own evolution and host factors, resulting in persistently low-level expression of serum HBsAg. This is also the difference between them.

## 3. Formation Mechanism of Persistent Low-Level HBsAg Expression

### 3.1. Virus Factors

HBV belongs to the hepatophilic DNA virus family. Due to the special covalent closed circular DNA (cccDNA) structure of the virus genome, the existing treatment strategies cannot completely eradicate HBV [25]. As long as there is a small amount of DNA residue, it can be used as a template for virus replication, causing HBV recurrence. The whole HBV gene consists of four partially overlapping open reading frames (ORFs): the pre-S/S region, pre-C/C region, P region, and X region. The pre-S/S region encodes three envelope proteins: large (L), medium (M), and small (S). The S protein corresponding to HBsAg is the main envelope protein of HBV. The pre-C/C region encodes nucleocapsid proteins (HBcAg and HBeAg). The DNA polymerase encoded by the P region is a multifunctional protein with reverse transcriptase (RT) and DNA polymerase activities. The regulatory protein X encoded by the X region can directly or indirectly regulate the expression of the host and virus genomes [26]. Due to the lack of proofreading function of the HBV polymerase, the high error rate occurs during HBV replication, resulting in the HBV genome being prone to mutation compared with other DNA viruses [27]. In addition, studies have shown that [28], in the case of HBeAg-negative chronic infection, the HBsAg level of HBV genotype D is significantly lower than that of genotypes A and E. In patients with genotype D infection, the specific mutation cluster at the C-terminus of HBsAg is associated with a decrease in HBsAg levels in vivo, hindering the release of HBsAg in vitro and affecting its structural stability.

There are a large number of HBV particles and viral proteins in patients with chronic HBV infection, and a variety of interactions can occur between viral proteins and the immune system. Some studies have shown that HBV replicates through RNA intermediates, which easily leads to mistakes, resulting in the rapid production of closely related but not completely identical virus variants, including virus variants that can escape the host immune response and antiviral treatment [29]. Meanwhile, a large number of deletion mutations accumulate in the genome of HBV mutant strains, which can further increase the complexity of chronic HBV infection. Moreover, HBV has a complete protein shell providing a strong self-protection ability, which is difficult to breach with current drugs. HBV mutates, enabling immune escape, when the drugs continue to act on a specific site. The base at that site will change, leading to drug resistance and rendering HBV treatment ineffective. Kirdar et al. [30] found that some chronic HBV-infected patients treated with nucleoside/nucleotide analogs have drug-resistant mutations in the HBV pol/S gene. This adaptive variation in HBV may cause the virus to achieve immune tolerance so that the host cannot remove low-level HBsAg from the serum. In addition, the increased incidence rate of HBsAg escape mutants also makes the virus escape vaccine-induced immunity, and these mutations make HBV infection management and screening more complex [31]. Actually, although manufacturers have improved the assay ability to detect HBsAg escape mutants using a mixed monoclonal/polyclonal combination to minimize unexpected missing HBsAg through epitope loss, the results vary considerably [32].

Furthermore, Graumann et al. [33] studied the effect of HBV genome-specific methylation on HBsAg expression and its role in HBsAg transgenic mice. The experimental results showed that the expression of HBsAg was negatively correlated with HBV DNA methylation. It is thought that the specific hypermethylation of cytosines in CpG dinucleotide acids in a specific HBV genome promoter region is related to the loss of HBsAg expression, which is the primary reason for the sharp decline in HBsAg content. Perumal et al. [34] found that HBV DNA methylation reduced the level of HBV mRNA and the expression of cell surface proteins and core proteins. Methylated HBV DNA could reduce the expression of HBsAg by up to 90%. Studies have shown that in the cell replication system or in the liver of chronic HBV-infected patients, HBV replication is indeed regulated by the acetylation state of H3/H4 histone bound to the viral cccDNA, and the hypoacetylation of histone is related to the reduction of HBV replication [35]. Therefore, the influence of histone modification cannot be ruled out during the formation of low-level HBsAg. Zhang et al. [36] verified by the GFP reporting method that miR-199a-3p and miR-210 directly affect the transcription of HBV RNA and inhibit the expression of HBsAg. Potenza et al. [37] also found that miR-125a-5p can interfere with the expression of HBsAg and reduce the secretion of HBsAg. In addition to direct targeting, some cell miRNAs can also regulate HBV replication by regulating cell epigenetic factors or specific transcription factors and then affect the secretion of HBsAg. Regarding the persistent low-level HBsAg expression of the host caused by the virus itself, the research on the relationship between P, X gene mutations and the formation of low-level HBsAg expression is not clear yet, and the following mainly introduces the persistent low-level HBsAg expression of the host caused by mutation of the S, pre-S, and C genes, as shown in Table 1.

#### 3.1.1. S Gene Mutation

S gene mutation leads to antigenicity changes, reduces the affinity between HBsAg and anti-HBs, and makes the value of HBsAg in serum decrease relatively (its absolute concentration does not decrease). The “a” determinant (aa124-147) in the major hydrophilic region (MHR, aa99-169) of the HBV S gene is an important antigen epitope that stimulates B cells to produce neutralizing antibodies. The site mutation on the “a” determinant can change its conformation and destroy the stability of the configuration to change its antigenicity and immunogenicity and affect its recognition by neutralizing antibodies. The antigen cannot be detected by conventional reagents, of which the sG145R mutation is the most classical S gene mutation [38, 39], which reduces the binding capacity of HBsAg to anti-HBs in the serum of hepatitis B vaccine recipients [40]. Other studies have found that the N-glycosylation modification of HBsAg MHR may interfere with the recognition of HBsAg by anti-HBs by masking B cell epitopes, resulting in the reduction of anti-HB binding affinity [41, 42]. Xiang et al. [43] found that mutations such as sL21R, sL95 W, and sL98 V outside the “a” determinant can also reduce the binding force between HBsAg and anti-HBs and affect the level of HBsAg inside and outside cells. Mutations inside/outside the “a” determinant may change the conformation of the “a” determinant and affect the modification of antigen protein, resulting in a decrease in its binding ability to antibodies and the production of immune escape or immune tolerance. Partial site mutation of the S gene can reduce the expression and secretion of HBsAg (absolute level decreased). Wu et al. [44] studied the effect of natural amino acid substitution on HBsAg by immunizing BALB/c mice. The experimental results confirmed that sT123N and sQ129 N substitutes effectively hindered the production of virus particles. Other studies have found that mutations such as sC124Y, sS136P, sC139R, sT140I, sK141E, and sD144A in the “a” determinant significantly reduce the secretion of virus particles, resulting in low-level or occult infection by HBV [45, 46]. In addition, some mutations in the S gene may also lead to early termination of transcription so that the virus cannot successfully synthesize and secrete HBsAg, like rtA181T/sW172, rtV191I/sW182, and rtM204I/sW196 mutations [47, 48]. The decrease in HBsAg secretion caused by partial site mutation of the S gene may be one of the formation mechanisms of low-level HBsAg in serum, which needs to be verified by simulating the expression of low-level HBsAg in serum in vitro.

#### 3.1.2. Pre-S Gene Mutation

HBV pre-S/S gene mutations are related to vaccine-induced immunity or immune escape of hepatitis B immunoglobulin [49]. The pre-S gene consists of pre-S1 (nt2848-3204) and pre-S2 (nt3205-154). The pre-S1 region contains many important HBV functional sites (S promoter, CCAAT binding factor, and nucleocapsid binding site). Deletion and point mutations of pre-S1 will lead to abnormal HBV synthesis and secretion. The pre-S2 region contains multiple recognition sites of T cells and B cells [50]. Pre-S1/S2 deletion mutations cause the virus to lose immune response-related epitopes, which affect the recognition of antigens by T cells or B cells, and the virus evades host immune surveillance, causing it to exhibit low-level replication in the host and establish persistent infection [51]. Pre-S deletion mutations are mainly concentrated in the 3′ end of pre-S1 and the 5′ end of pre-S2, and pre-S2 deletion mutations are the most common. Pollicino et al. [52] analyzed the replication ability of 40 chronic HBV patients with the pre-S mutation and found that the 183 bp deletion mutation in the pre-S1 region and HBV replication ability decreased in patients with deletion of the pre-S2 start codon. Our research group analyzed the pre-S sequence of the low-level HBsAg population in patients with chronic HBV infection and found that the mutation frequencies of L34F, V49A, F56I/V, P59S/L, T76A/N/P, W66 V/G, A79 V in the pre-S1 region and V32A and T49I in the pre-S2 region were higher than those in high-level HBsAg population (*P* < 0.05) [53]. The findings of the above studies suggest that deletion and point mutations in the pre-S region have a certain correlation with low-level HBsAg expression in serum [54].

#### 3.1.3. C Gene Mutation

The HBV C gene is divided into the pre-C gene region and the C gene region, encoding HBeAg and HBcAg, respectively. The most frequently detected mutation in the pre-C region in HBV-infected people is the nucleotide (nt) 1896 mutation, which changes guanine (G) to adenine (A) and the tryptophan codon (TGG) to a stop codon (TAG), resulting in the termination of HBeAg synthesis. Serum HBeAg positivity is a sign of HBV replication ability and strong infectivity, while HBsAg quantification and HBV DNA viral load in HBeAg-negative patients are lower than those in HBeAg-positive patients [55]. In addition, the basic core promoter (BCP) region adjacent to pre-C is the key to the initiation of HBV replication. In a study of BCP/PC mutations in a mouse model, Liu et al. [56] found that HBV BCP/PC mutations are related to low levels of HBsAg in serum and can lead to an inefficient immune response in the late stage of chronic HBV infection.

### 3.2. Host Factors

HBV mainly completes replication in liver cells. To completely inhibit HBV replication, the drug must be able to efficiently enter liver cells. The function of the liver to induce immune tolerance is unique [57]. Antigens that are continuously expressed in the liver often induce local or systemic immune tolerance rather than an immune response. The physiological structure, cell composition, and other characteristics of the liver provide a time and space basis for the liver to induce immune tolerance to achieve systemic immune tolerance through a variety of mechanisms, such as inducing T cell clonal clearance, clonal incompetence, immune deviation, and inducing immune negative regulatory cells [58, 59]. Thus, HBV exploits the immune tolerant environment of the liver, which may lead to persistent virus infection and rapid development of cancer [60]. Other studies have shown that adaptive immune cells are more tolerant in the liver than in other organs [61], and there is a relationship between the adaptive immune response and the liver tolerance microenvironment [62]. This tolerant microenvironment can lead to liver T cell dysfunction, including clonal deletion, nonresponse, aging, deviation, and failure [63]. The liver contains a large number of hepatocytes, nonparenchymal cells, and lymphocytes, and the complex interactions among these cells help induce adaptive immune tolerance in the liver [64]. Studies have confirmed that intrinsic antigen-presenting cells (APCs) in the liver can induce immune tolerance to allow the virus to escape immune clearance or mediate an effective antiviral immune response against HBV infection [65]. It was also previously reported that HBV DNA was detected in extrahepatic tissues such as the kidney, spleen, and pancreas [66]. These organs may not cause an effective immune response, and thus, the virus can escape host immune clearance, resulting in repeated release of the virus, the formation of an extrahepatic virus repository, and repeated infection of hepatocytes, resulting in chronic infection.

A circulating immune complex (CIC) is formed by an antigen and its specific antibody binding together during an immune response. Under normal circumstances, CIC formation is a way for the body to clear antigens, and it has a protective effect on the body. However, under pathological conditions, CIC can cause an immune response that is too strong and directly damages hepatocytes. Some studies have analyzed the difference in serum CIC between high-level and low-level HBsAg populations in patients with chronic HBV infection. Some HBsAg in the serum of the low-level HBsAg population exists in the form of CIC (HBsAg CIC), and the specific CIC positive rate is relatively high. Because the body is in a low response state for a long time, the body's ability to clear CIC decreases. As a result, the concentration of free HBsAg in serum decreases or cannot even be detected [67]. At the same time, the use of immunosuppressants is also related to a low-level state of HBsAg. Because the use of immunosuppressants enhances the body's ability to escape the immune response, it is unable to completely eliminate HBsAg, resulting in low-level expression of HBsAg in the body [68].

Moreover, it has been reported that the ethnicity, age, sex, and education level of the host were significantly correlated with the status of HBV infection [69]. It has been found that the age of HBV-infected persons with low levels of HBsAg is relatively high (>50 years old) [70], and a sustained low level of HBsAg expression may be related to the status of inactive HBV carriers and may be associated with increasing age. Yuen et al. [71] also showed that HBV variation is related to an increase in age and a decrease in HBsAg levels. An increase in HBV diversity may lead to the virus escaping immune surveillance and forming immune tolerance, resulting in a sustained low level of HBsAg expression in serum. In addition, WHO advocates administration of the hepatitis B vaccine as soon as possible after birth [72], and in some low-income and middle-income countries, due to a lack of resources, HBV's detection and diagnostic techniques are limited, resulting in decades of immune tolerance due to a lack of medical care. Although the HBV vaccine has reduced the incidence of HBV, it will still allow vertical transmission of HBV in the area. Therefore, chronic HBV infection in these areas may be more likely to lead to immune tolerance, resulting in a low level of serum HBsAg expression [73].

**Figure 1:**
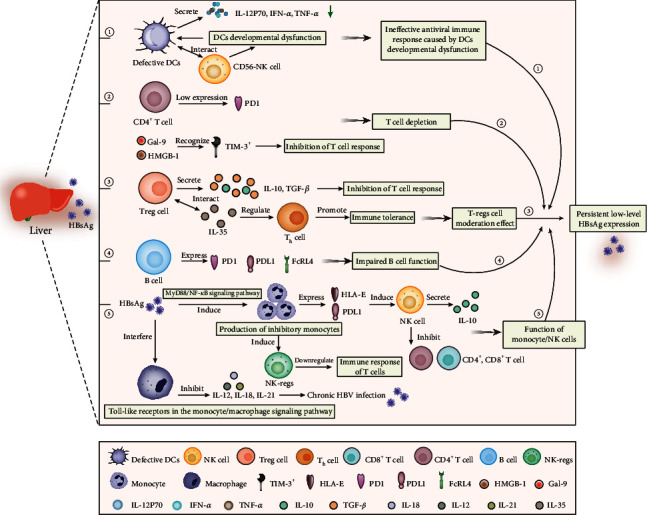
Host immunity mediated by immune cells in liver tolerance microenvironment leads to persistent low-level HBsAg expression in patients with chronic HBV infection: (1) Dysplasia of DCs leads to inefficient antiviral responses; (2) Continued expression of coinhibitory receptors maintains T cell exhaustion; (3) T-reg regulation suppresses T-cell responses leading to immune tolerance; (4) Dysfunctional B cells express coinhibitory receptors; and (5) Immunosuppressive monocytes/NK cells play a role in inhibiting T cell response.

Through the above discussion, it can be found that the low-level expression of HBsAg in serum is a relatively stable and complex dynamic balance between virus replication and host clearance under the action of various factors [74]. Among them, dysfunction of the host immune system and the development of immune tolerance are considered to be the key factors in the formation of sustained low-level HBsAg, which has become a research hotspot and a breakthrough point in HBV therapy. The host immune system is an important factor affecting the prognosis after HBV infection. During HBV infection, the immune response produced by the body is jointly regulated by a variety of immune cells (such as dendritic cells (DCs), T/B lymphocytes, monocytes/natural killer (NK) cells, and immune factors (IFN, TNF, and IL) [75]). When the number of immune cells decreases or the immune regulation function of cytokines is defective, the immune response of the body is weakened, the immune function is disordered, and immune tolerance develops, resulting in the host's inability to effectively remove the virus, which leads to chronic progression of the disease [76]. A persistent low level of HBsAg in the host serum may not stimulate the host to produce an immune response or only a low response. A long-term nonresponse or low response state may lead to the host forming immune tolerance against a persistent low level of HBsAg expression, so it is impossible to completely eliminate HBV through humoral immunity or cellular immunity. Since there are few reports on the relationship between humoral immunity and the development of persistently low levels of HBsAg expression, this paper mainly summarized the host immunity mediated by DCs, T/B lymphocytes, and monocytes/natural killer cells in the liver-tolerant microenvironment, as shown in Figure 1.

#### 3.2.1. Dendritic Cells

As an APC, DCs can absorb and process HBsAg and secrete a variety of cytokines to participate in the regulation of immune tolerance and immune responses in patients with chronic HBV infection [77]. At the same time, the liver contains different nonparenchymal cell populations, which can also be used as APCs. When the starting efficiency of the effector T cells is low, they tend to induce T cell tolerance rather than T cell activation [78]. It has been reported that DC developmental dysfunction in the peripheral blood of chronic HBV-infected patients, serum HBsAg, IFN-*α*, TNF-*α*, and other cytokine levels have been reduced to varying degrees [79], and defective DCs and CD_56_ NK cells may lead to an ineffective antiviral immune response and the formation of immune tolerance [80]. In addition, studies by Yang et al. [81] have shown that the cytokine IL-35 regulates the function of regulatory T cells (T-regs) and T helper (Th) 17 cells during chronic HBV infection, which may contribute to immune tolerance and virus persistence. Therefore, in a case of chronic HBV infection and impaired DC function, the virus may escape and establish a low level HBsAg hepatitis B virus [82], and DCs cannot successfully absorb and process low-level HBsAg, resulting in the body being unable to produce an effective immune response to low-level HBsAg, and can create immune tolerance, which leads to the persistence of low-level HBsAg.

#### 3.2.2. T/B Lymphocytes

Chronic HBV infection is associated with T cell depletion [83], which is characterized by sustained expression of coinhibitory receptors and impaired cytokine production, such as programmed death receptor-1 (PD-1), natural killer cell receptor 2B4 (CD_244_), cytotoxic T lymphocyte-associated antigen-4 (CTLA-4), T cell immunoglobulin and mucin-3 (Tim-3), and cytokine CD_160_. High levels of HBsAg in serum can induce the upregulation of coinhibitory receptors, and the continuous expression of a variety of coinhibitory receptors can maintain the functional failure of T cells. Ganjalikhani et al. [84] found that in chronic HBV infection, the coinhibitory receptor Tim-3^+^, which is recognized by ligands such as C-type lectin galectin-9 (Gal-9) and high mobility group protein-1 (HMGB-1), may inhibit the T cell response in the liver microenvironment and then promote persistent HBV infection. At the same time, T-regs, as a special subgroup of CD4^+^ T cells, secrete a variety of effector molecules, such as IL-10 and TGF-*β*, when HBV infects a host, and directly or indirectly inhibit the T cell response to maintain immune tolerance [85]. However, studies have shown that compared with patients with high serum levels of HBsAg, patients with low serum levels of HBsAg have lower PD-1 expression on CD4^+^ T cells and they downregulate the expression of PD-1, which may provide critical support for the virus clearance of chronic HBV infection, but it has also been reported that there is no significant difference in 2B4 expression on CD4^+^ T cells [86, 87]. Therefore, persons with chronic HBV infection with low-level HBsAg present an inactive HBsAg-carrying state. Although some coinhibitory receptors in the body are low, T cell depletion may still exist, resulting in incomplete recovery of T cell function, an inability to respond normally to low-level HBsAg and immune tolerance, so that HBV cannot be completely cleared. It has also been reported that when a large number of viruses infect the host, if the T cell response is not very strong, even if the host produces antiviral cytokines, cytotoxic T lymphocytes cannot completely inactivate HBV, which will eventually lead to continuous infection with the virus [88]. In other words, in the case of an incomplete T cell response, low-level HBsAg may escape immune surveillance, resulting in weak viral gene expression in the host so that HBV cannot be completely cleared [89]. In addition, B cells associated with dysfunction and failure also express a series of coinhibitory receptors, including Fc receptor like 4 (FcRL4), PD-1, and PD-L1 [90], although there was no significant difference between the low-level HBsAg group and the high-level HBsAg group, B cell function in hepatitis B patients with low levels of HBsAg may still be impaired, resulting in immune tolerance [86]. Therefore, in conclusion, low-level HBsAg may induce complete or incomplete immune tolerance of host T and B lymphocytes, resulting in the host's failure to completely eliminate low-level HBsAg.

#### 3.2.3. Monocyte/Natural Killer (NK) Cells

Host immunity mediated by NK cells and monocytes is an important part of the innate immune response. Studies have found that NK cells in patients with high serum HBsAg and chronic HBV infection have functional defects and are inactivated [91]. Li et al. [92] observed that HBV could induce the production of inhibitory monocytes by HBsAg through a variety of signaling pathways. Human leukocyte antigen-E (HLA-E) and PD-L1 expressed by these inhibitory monocytes can further induce NK cells to produce IL-10. Such NK cells can inhibit the activation of host CD4^+^ and CD8^+^ T cells, and HBV can also induce regulatory NK cells (NK regs) through monocytes to downregulate the immune response of T cells. At the same time, it has been reported that NK cells in patients with chronic HBV infection can mediate immune tolerance or immune injury [93]. Other studies have shown that HBsAg can interfere with toll-like receptors (TLRs) in the monocyte/macrophage signaling pathway to inhibit the release of cytokines, which may help to establish a chronic infection [94, 95]. However, Kim et al. [86] have shown that there is no significant difference in the composition of immune cells between monocytes and NK cells in the serum low-level HBsAg group and serum high-level HBsAg group. Therefore, chronic HBV infection may cause monocyte and NK cell dysfunction due to long-term HBV infection and block the normal recognition and killing effect of monocytes and NK cells. They cannot effectively release anti-inflammatory and toxic inflammatory factors and completely inhibit HBV, resulting in the persistence of low-level HBsAg in the serum.

## 4. Conclusions

In summary, low-level HBsAg is a persistent low-level state accompanied by low or no HBV DNA replication, distinct from occult hepatitis. Furthermore, there are various reasons for the formation of continuous low-level HBsAg expression in patients with chronic HBV infection, which is generally due to the host's failure to completely eliminate low-level HBsAg under the various effects of viral, host and other factors. At present, the research reports on the formation mechanism of the sustained low-level expression of HBsAg in patients with chronic HBV infection are not comprehensive. However, patients with low-level HBsAg still have virus replication and sustained liver injury, and they may spread infection, with the continuous development of HBV diagnosis and treatment technology, the low-level HBsAg population has attracted increasing attention from experts in the fields of hepatology, infectious disease, and epidemiology. Therefore, studying the mechanism underlying continuous low-level HBsAg expression in serum will lay the foundation for breaking immune tolerance after HBV infection in clinical treatment to expand new ideas for the complete elimination of HBV.

## Figures and Tables

**Table 1:** Persistent low level of HBsAg in serum of patients with chronic HBV infection caused by viral factors.

Viral factors	Classification	Mechanism	Amino acid changes	Reference
HBV genotype	Genotype D	In genotype D, the specific mutation cluster at the C-terminus of HBsAg hindering the release of HBsAg in vitro and affecting its structural stability.		[28]
Epigenetic changes	HBV DNA methylation	Reduce the level of HBV mRNA and the expression of cell surface protein and core protein.		[33, 34]
	Histone hypoacetylation	Hypoacetylation of histones is related to the reduction of HBV replication.		[35]
	miRNA feedback mechanism	miRNAs affect the replication and transcription of HBV, thereby inhibiting the expression of HBsAg.	miR-199a-3p, miR-210, miR-125a-5p	[36, 37]
HBV gene mutation	S gene			
	“a” determinant	Lead to antigenic changes and reduce the affinity between HBsAg and anti-HBs.	sG145R	[38–40]
		Partial site mutation of S gene reduces the expression and secretion of HBsAg.	sC124Y, sQ129R, sM133T, sS136P, sI126S/T, sC139R, sT140I, sD144A/E, sG145R/K, sK141E	[45, 46]
	Outside the “a” determinant	Lead to antigenic changes and reduce the affinity between HBsAg and anti-HBs.	sE2G, sC69stop, sL95W, sL21S, sR24K, sL98V, sG119R, sK122I, sT123N, sA159G	[43, 44, 46]
		Partial site mutation of S gene reduces the expression and secretion of HBsAg.	sE2G, sL95W, sL98V	[40, 43]
		The transcription of some mutations in the S gene is terminated prematurely, making the virus unable to successfully synthesize and secrete HBsAg.	sW172stop, sW182stop, sW196stop	[47, 48]
	Pre-S gene			
	Pre-S1 deletion mutation	Reduction of virus replication and secretion.	Pre-S1L34F, pre-S1V49A, pre-S1F56I/V, pre-S1P59S/L, pre-S1W66V/G, pre-S1T76A/N/P, pre-S1A79V, pre-S1 fragment deletion	[52–54]
	Pre-S2 deletion mutation	The loss of immune-related epitopes reduces the recognition ability of T cells to antigens.	Pre-S2Q2K, pre-S2V32A, pre-S2T49IMlI, pre-S2 fragment deletion	[50, 52, 53]
	C gene	Mutations in the BCP/PC regions of HBV are related to low serum HBsAg levels, which can lead to ineffective immune response in the late stage of chronic HBV infection.	nt1896	[55, 56]

## Abbreviations

HBsAg: Hepatitis B surface antigen
HBcAg: Hepatitis B core antigen
IT: Immune-tolerant
IC: Immune clearance
LR: Low-replicative
ENH: HBeAg-negative hepatitis
ELISA: Enzyme-linked immunosorbent assay
CMIA: Chemiluminescence microparticle immunoassay
OBI: Occult hepatitis B infection
ORFs: Open reading frames
MHR: Major hydrophilic region
BCP: Basic core promoter
APCs: Antigen-presenting cells
CIC: Circulating immune complex
DCs: Dendritic cells
NK: Natural killer
TNF: Tumor necrosis factor
T-regs: Regulatory T cells
PD-1: Programmed death receptor-1
CTLA-4: Cytotoxic T lymphocyte-associated antigen-4
Tim-3: T cell immunoglobulin and mucin-3
Gal-9: C-type lectin galectin-9
HMGB-1: High mobility group protein-1
FcRL4: Fc receptor like 4
HLA-E: Human leukocyte antigen-E
TLR: Toll-like receptor.
